# Supplementary material for: A light-fueled self-oscillator that senses force
Source: Commun Mater. 2025 Aug 5;6(1):173. doi: 10.1038/s43246-025-00903-2 (PMC12325081; doi:10.1038/s43246-025-00903-2)
Supplement: Supplementary file 2 — Supplementary Information [file 43246_2025_903_MOESM2_ESM.pdf]

Supplementary Materials for

A Light-Fueled Self-Oscillator That Senses Force

Zixuan Deng[1], Arri Priimagi[1], Kai Li[2], Hao Zeng[1]**

[*]E-mail: kli@ahjzu.edu.cn, hao.zeng@tuni.fi.

This PDF file includes:

1. **Supplementary Figures 1-17.**
2. **Captions for Supplementary Movies 1-2.**

1. Supplementary Figures.

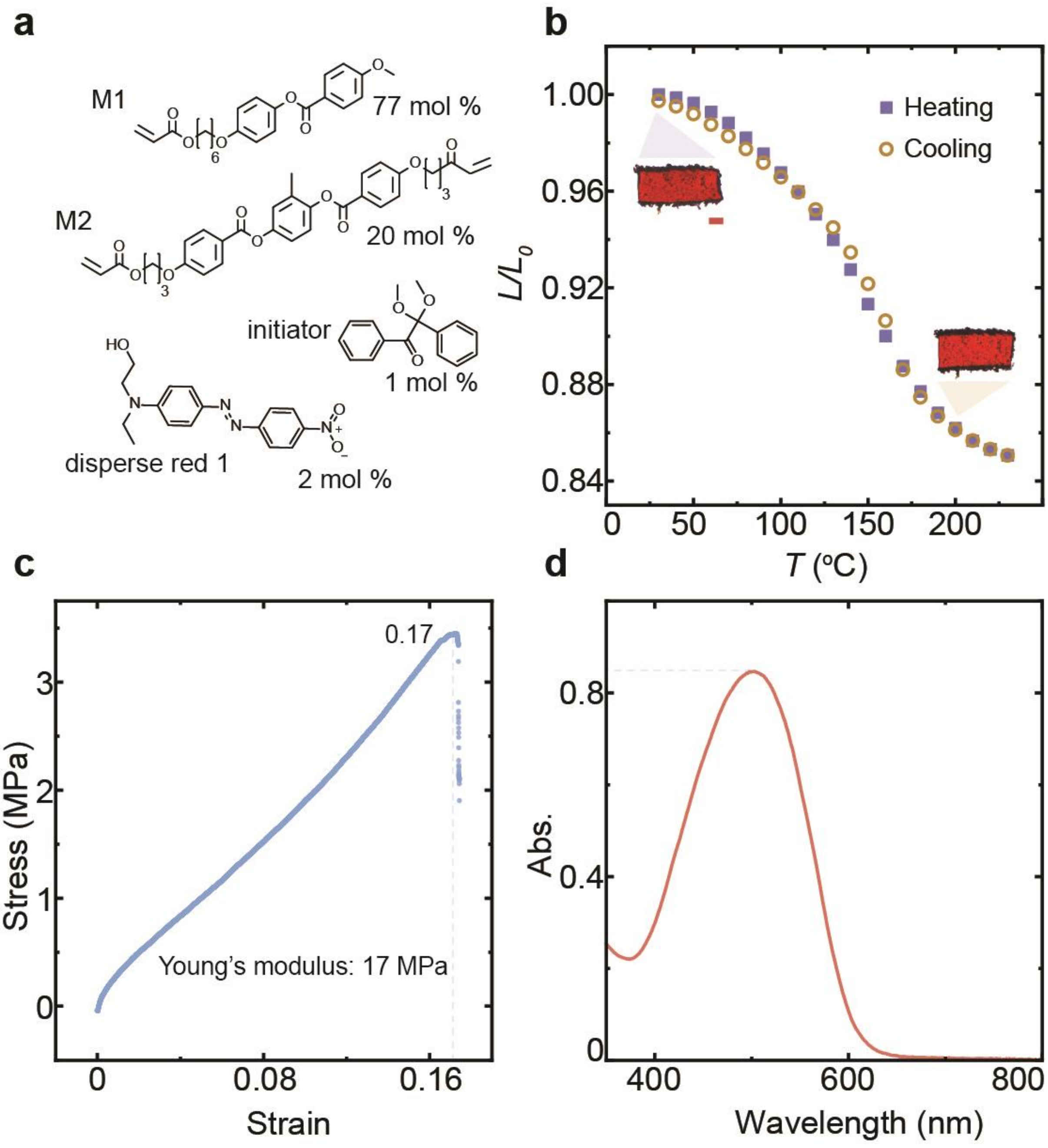

Supplementary Figure 1. Material study. a) Compounds used for the fabrication of light-responsive LCN. **b)** Heat-induced deformability in a planar-aligned LCN sample along the alignment direction. L_0: original length along the director. L: sample length after deformation. Insets: images of LCN sample deforming upon heating at 30°C (left) and 200°C (right). Scale bar: 200 µm. **c)** Mechanical testing of an LCN strip, with stretching along the alignment direction. **d)** Absorption spectrum of a 10 µm thick LCN film.

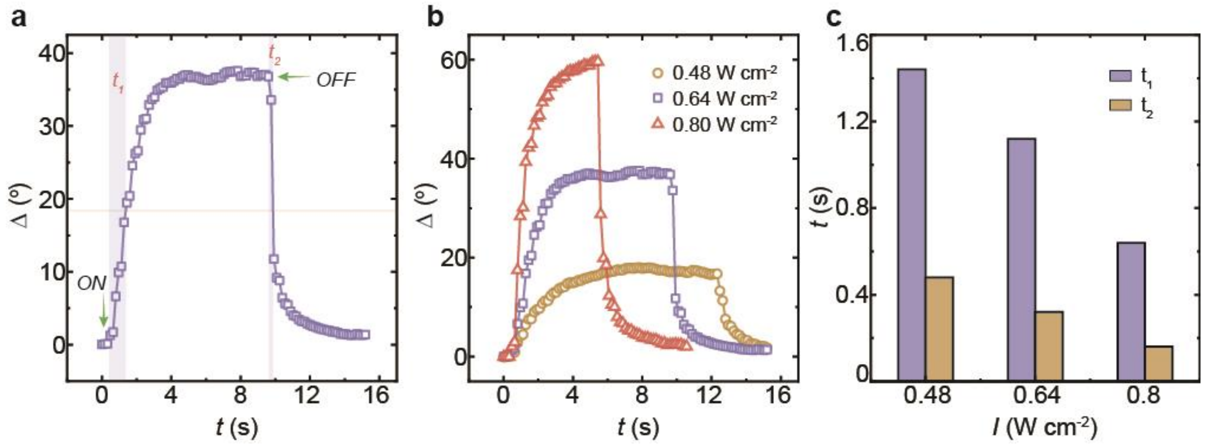

Supplementary Figure 2. Deflection kinetics. a) Planar-aligned LCN deflecting under light illumination. t_1 represents the duration from the pristine state to the half of the maximal deformation state. t_2 represents the duration from the maximal deformation state to its half state after ceasing the light irradiation. Irradiation conditions: 532 nm, 0.64 W cm^{-2}. **b)** Deflection angle of a planar LCE strip exposed to different light intensities. **c)** t_1 and t_2 for different light intensities. Irradiation conditions: 532 nm.

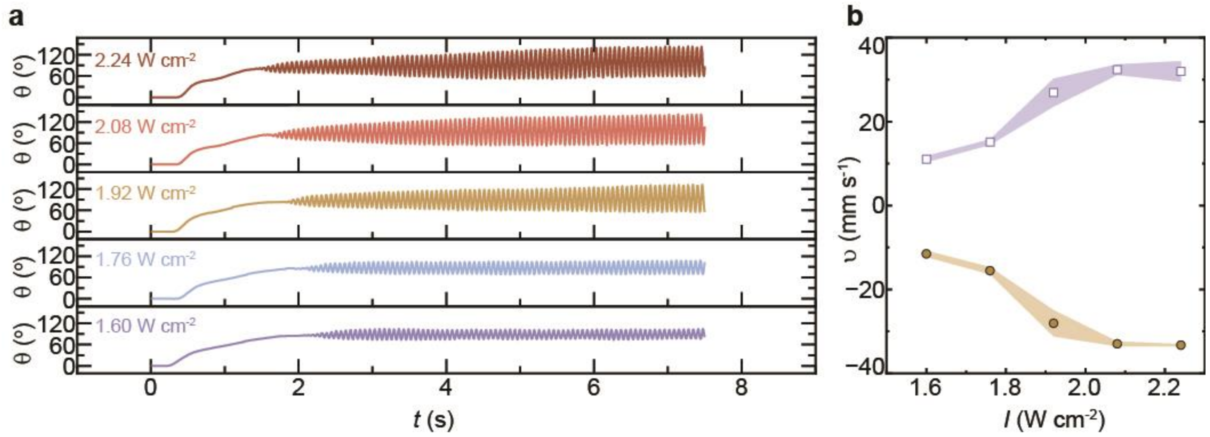

Supplementary Figure 3. Self-shadowing kinetics. a) Time-resolved oscillation angle of the planar-aligned LCN strip under different intensities. **b)** Corresponding maximal oscillation velocity for different intensities. Irradiation conditions: 532 nm. The error bars in (b) are displayed as mean values +/- standard deviation (n = 3). The same sample was measured repeatedly.

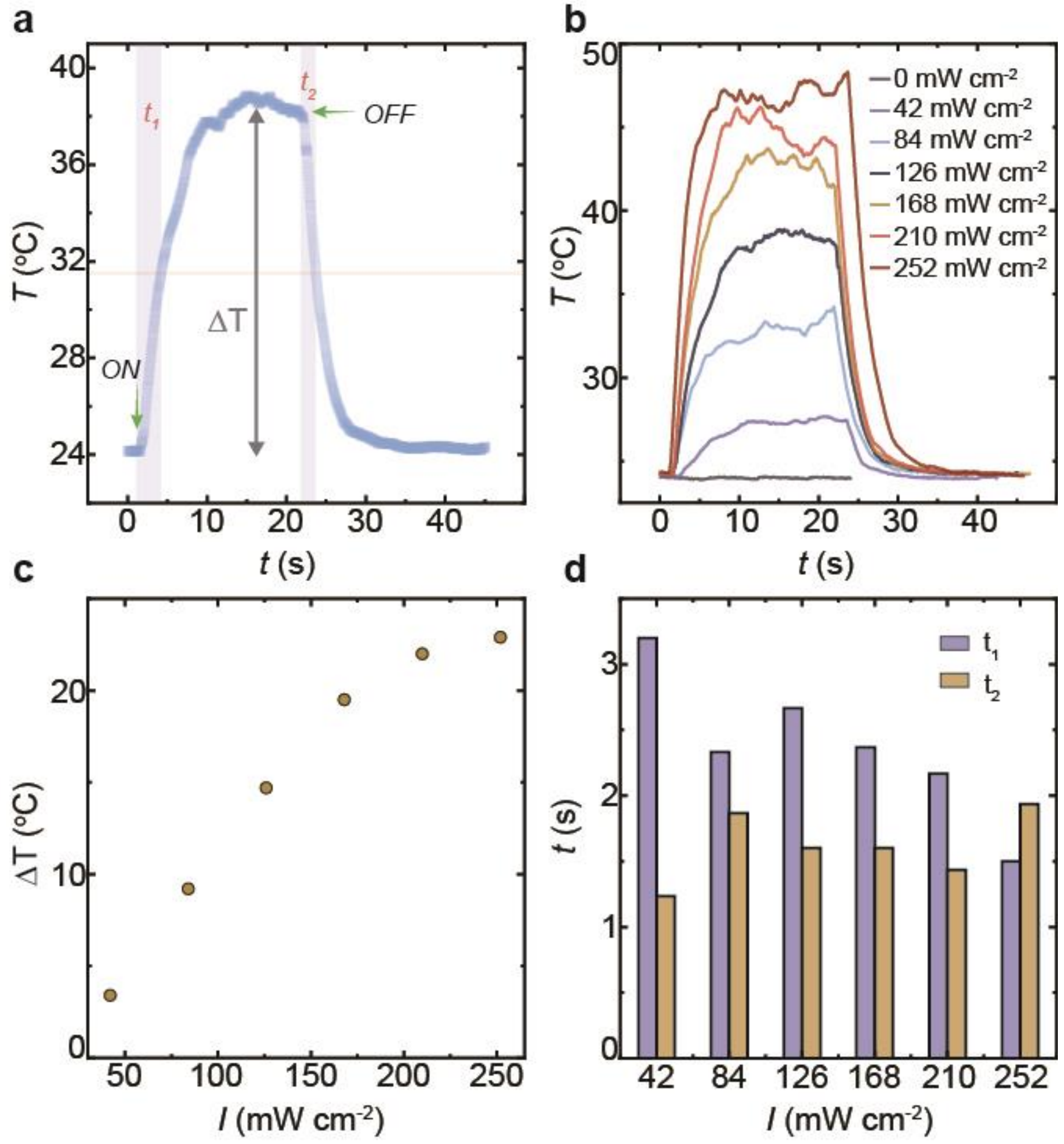

Supplementary Figure 4. Kinetics of thermal profiling. a) Time evolution of the temperature in a planar-aligned LCN strip under external load, under large-area light illumination. t_1 represents the duration from the pristine state to the half of the maximal temperature. t_2 represents the duration from the maximal temperature to its half state after ceasing the light irradiation. Irradiation conditions: 532 nm, 126 mW cm^{-2}. **b)** Temperature elevation of a planar LCE strip exposed to different light intensities. Load condition: 2.4 g weight. **c)** Temperature difference (*ΔT*) to room temperature (24°C) for different light intensities. **d)** t_1 and t_2 for different light intensities. Irradiation conditions: 532 nm.

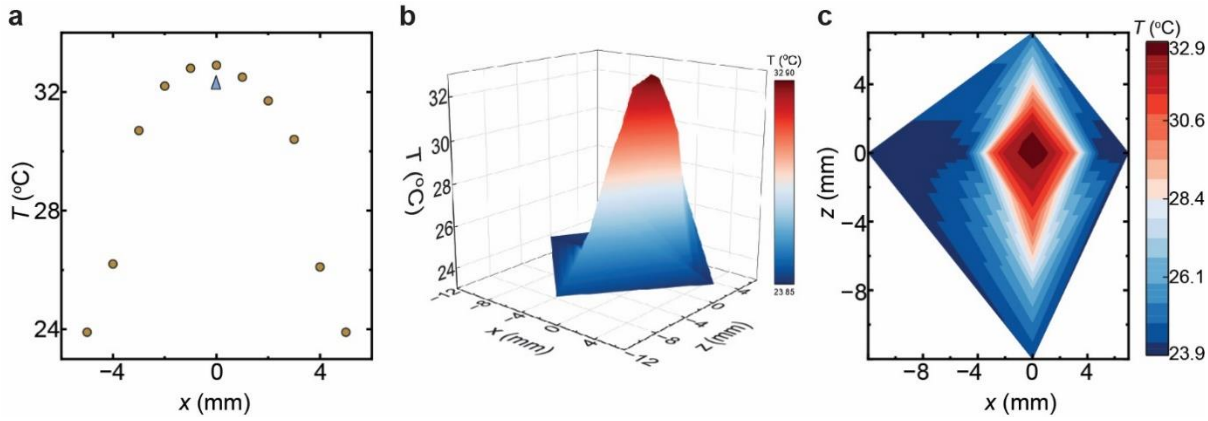

Supplementary Figure 5. Distribution of thermal imaging. a) Temperature distribution along the x-axis. **b)** 3D construction of the temperature distribution of a planar-aligned LCE strip and **c)** its projection in x-z plane. Load condition: 2.4 g weight. Irradiation conditions: 532 nm, 84 mW cm^{-2}.

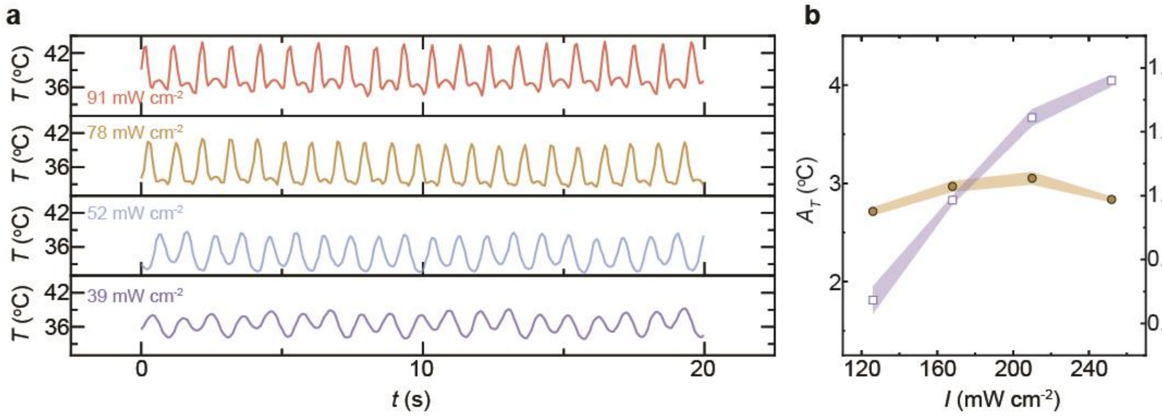

Supplementary Figure 6. Thermal oscillation kinetics. a) Time-resolved temperature fluctuations of the planar-aligned LCN strip under different intensities. **b)** Summary of temperature fluctuation amplitude and frequency for different intensities. Load condition: 2.4 g weight. Irradiation conditions: 532 nm. The error bars in (b) are displayed as mean values +/- standard deviation (n = 3). The same sample was measured repeatedly.

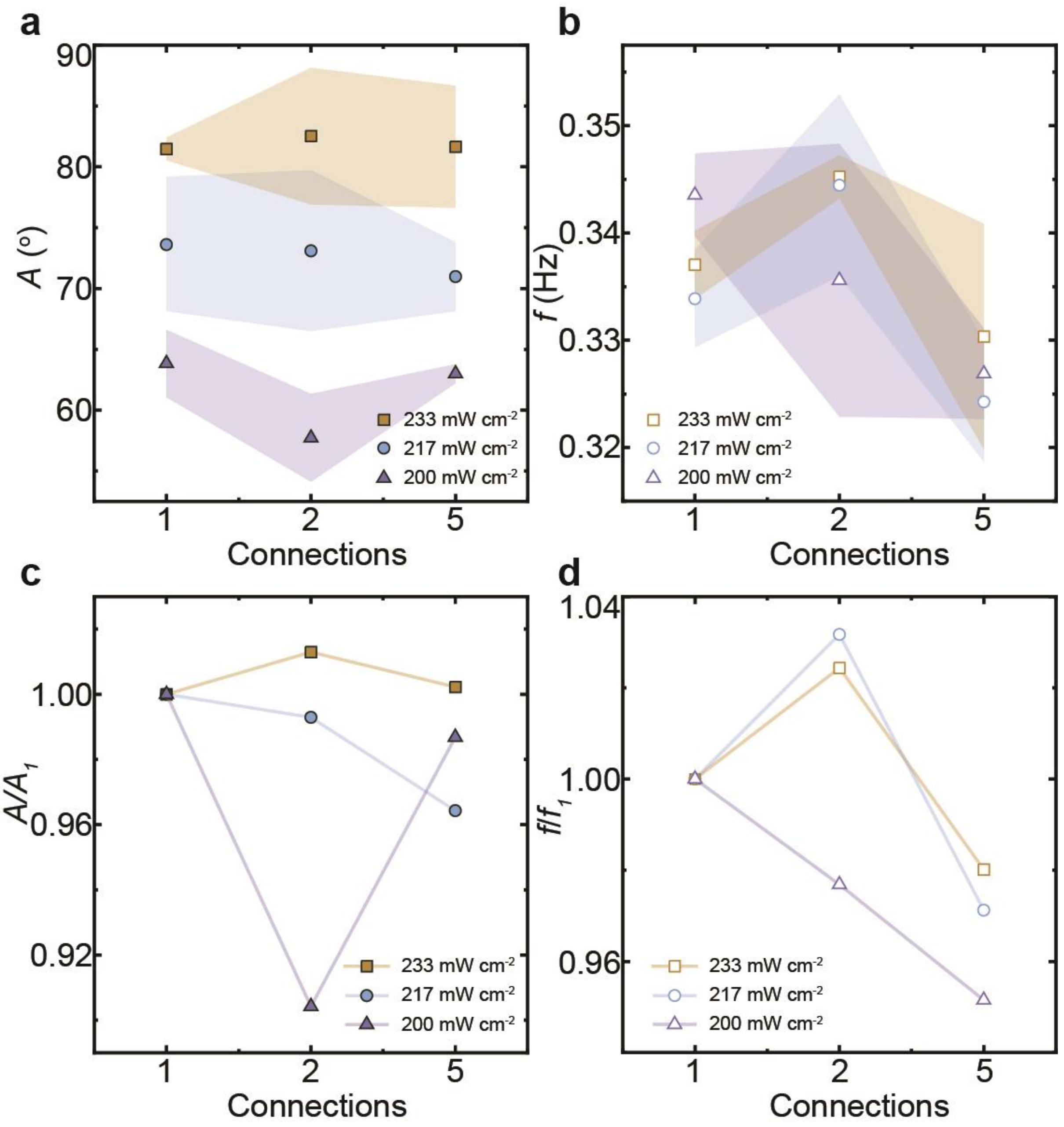

Supplementary Figure 7. Multi-thread connection. a) Oscillation amplitude and **b)** frequency as a function of the number of connection via 1, 2, 5 wires under three light intensities. The error bars are displayed as mean values +/- standard deviation (n = 3). The same sample was measured repeatedly. Relative variation in **c)** amplitude and **d)** frequency, normalized to the single-point contact case. A_1 and f_1 denote the amplitude and frequency for the single-point connection, respectively. Load condition: 2.4 g weight. Irradiation wavelength: 532 nm.

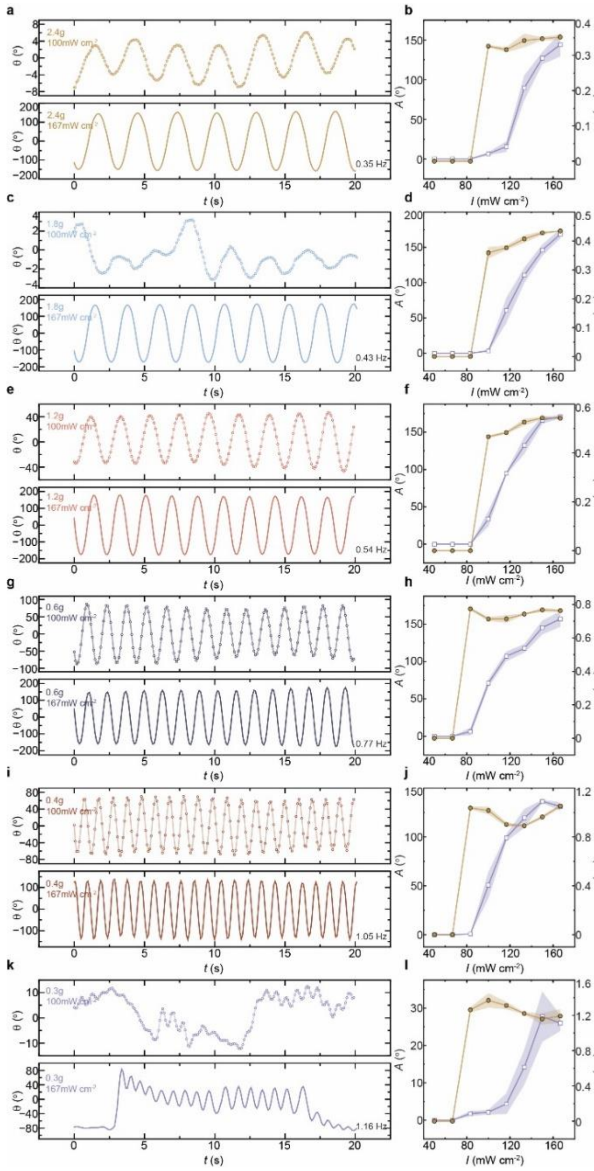

Supplementary Figure 8. Effects of intensity on oscillation in planar mode. a) Time-resolved rotation angle of the planar-aligned LCN strip, attached with 2.4 g load, under (top) 100 mW cm^{-2} and (bottom) 167 mW cm^{-2} illumination. **b)** Summary of oscillation amplitude and frequency for different intensities. **c)** Time-resolved rotation angle of the planar-aligned LCN strip, attached with 1.8 g load, under (top) 100 mW cm^{-2} and (bottom) 167 mW cm^{-2} illumination. **d)** Summary of oscillation amplitude and frequency for different intensities. **e)** Time-resolved rotation angle of the planar-aligned LCN strip, attached with 1.2 g load, under (top) 100 mW cm^{-2} and (bottom) 167 mW cm^{-2} illumination. **f)** Summary of oscillation amplitude and frequency for different intensities. **g)** Time-resolved rotation angle of the planar-aligned LCN strip, attached with 0.6 g load, under (top) 100 mW cm^{-2} and (bottom) 167 mW cm^{-2} illumination. **h)** Summary of oscillation amplitude and frequency for different intensities. **i)** Time-resolved rotation angle of the planar-aligned LCN strip, attached with 0.4 g load, under (top) 100 mW cm^{-2} and (bottom) 167 mW cm^{-2} illumination. **j)** Summary of oscillation amplitude and frequency for different intensities. **k)** Time-resolved rotation angle of the planar-aligned LCN strip, attached with 0.3 g load, under (top) 100 mW cm^{-2} and (bottom) 167 mW cm^{-2} illumination. **l)** Summary of oscillation amplitude and frequency for different intensities. Irradiation conditions: 532 nm. The error bars are displayed as mean values +/- standard deviation (n = 3). The same sample was measured repeatedly.

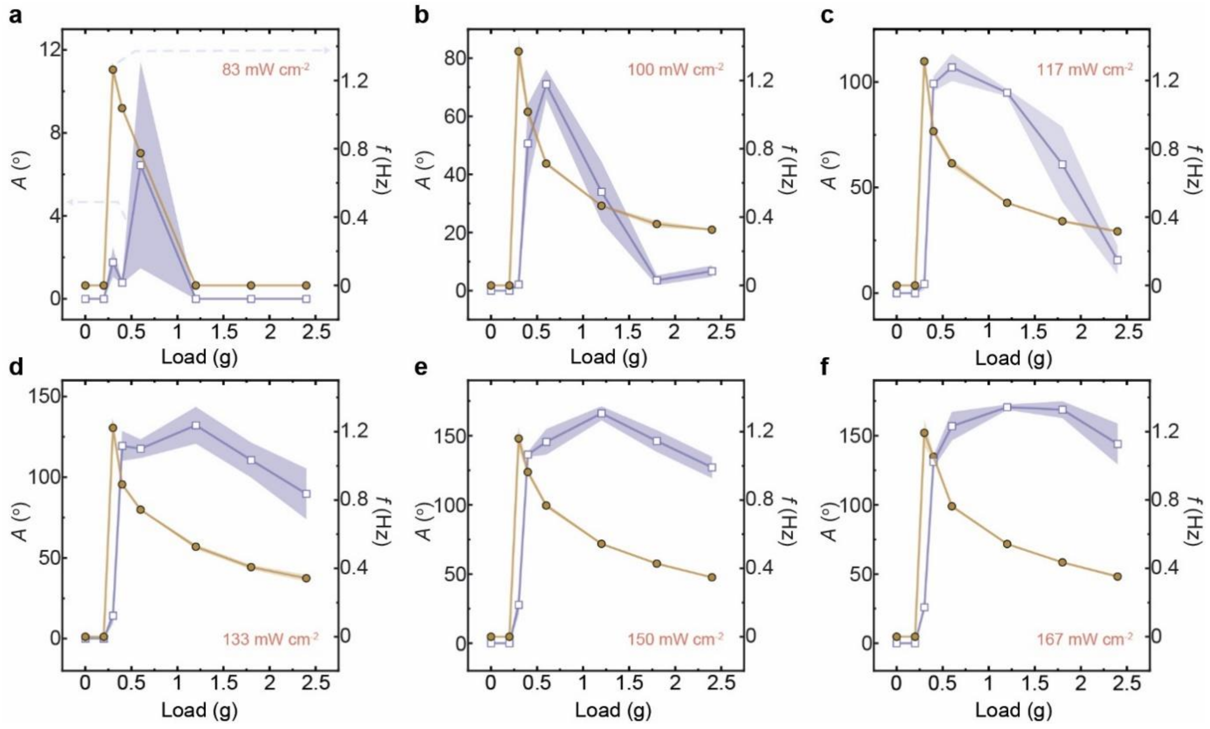

Supplementary Figure 9. Effects of weight of the load on oscillation in planar mode. a) – f) Summary of oscillation amplitude and frequency under varied loads for different intensities. Irradiation conditions: 532 nm. The error bars are displayed as mean values +/- standard deviation (n = 3). The same sample was measured repeatedly.

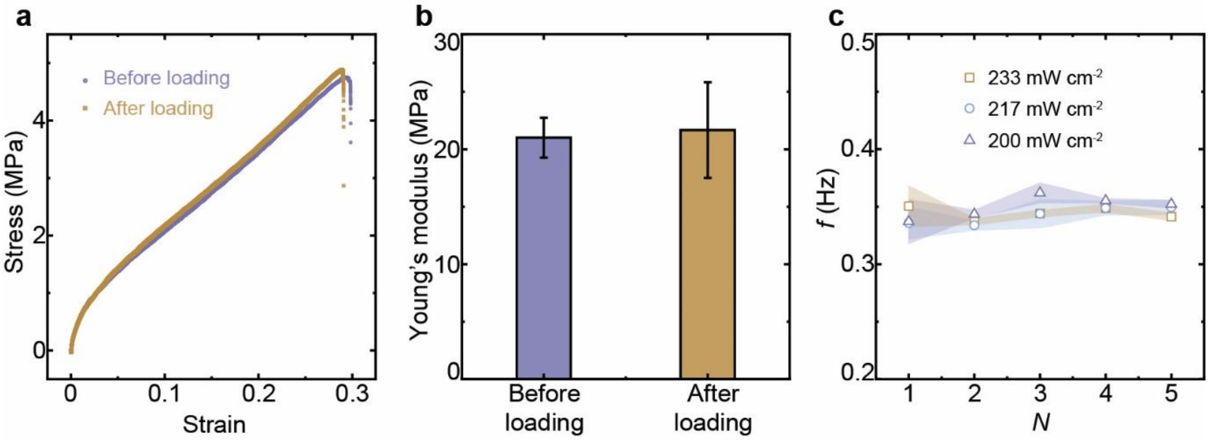

Supplementary Figure 10. Negligible effect of stretching on material properties. a) Mechanical testing of LCN strips stretched along the alignment direction before and after 4 hours of loading. **b)** Calculated Young's modulus before and after loading. The error bars are displayed as mean values +/- standard deviation (n = 3). **c)** Oscillation frequency under repeated loading–unloading cycles at three light intensities. *N* denotes the number of loading cycles. The error bars are displayed as mean values +/- standard deviation (n = 3). The same sample was measured repeatedly. Load condition: 2.4 g weight. Irradiation conditions: 532 nm.

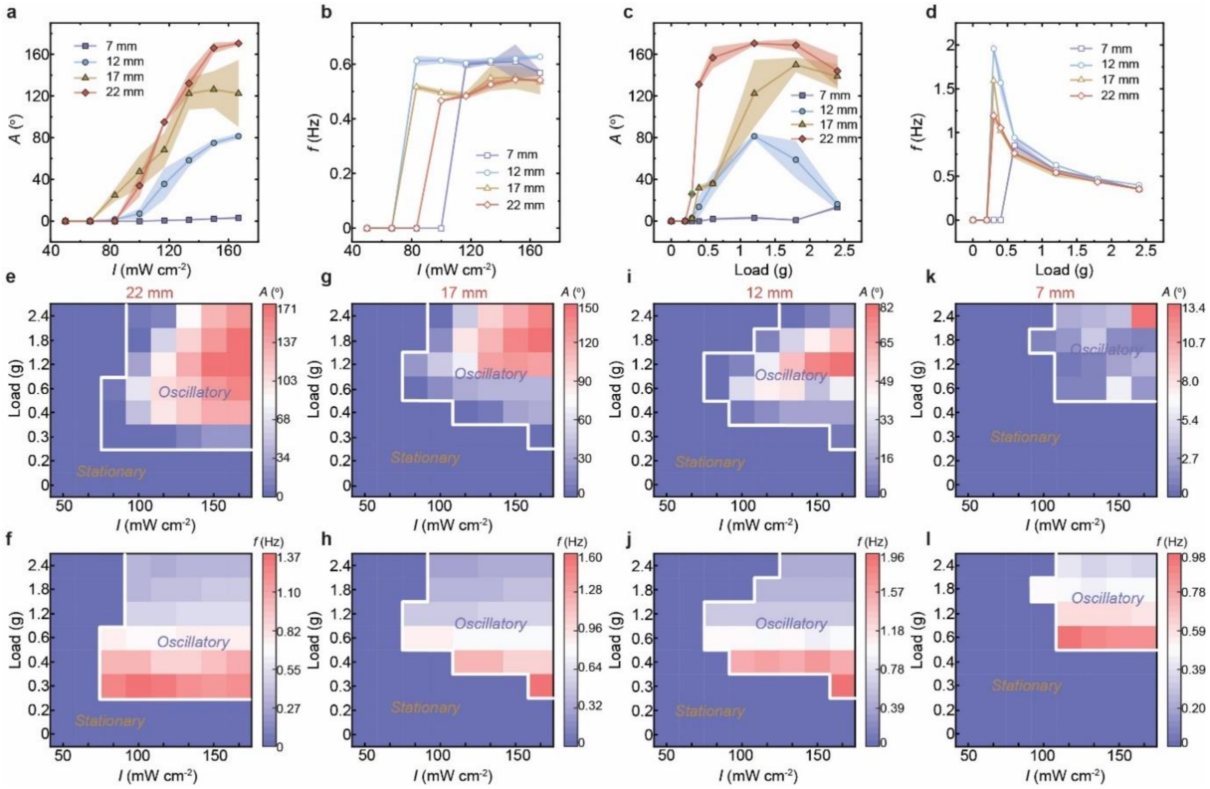

Supplementary Figure 11. Effects of excitation length on oscillation in planar mode. Summary of oscillation **a)** amplitude and **b)** frequency under varied intensities for different excitation lengths. Load condition: 1.2 g weight. Summary of oscillation **c)** amplitude and **d)** frequency under varied loads for different excitation lengths. Irradiation conditions: 532 nm, 167 mW cm^{-2}. Heat map illustrating the oscillation **e)** amplitudes and **f)** frequencies across a range of illumination intensities and attached loads at an excitation length of 22 mm. Heat map illustrating the oscillation **g)** amplitudes and **h)** frequencies across a range of illumination intensities and attached loads at an excitation length of 17 mm. Heat map illustrating the oscillation **i)** amplitudes and **j)** frequencies across a range of illumination intensities and attached loads at an excitation length of 12 mm. Heat map illustrating the oscillation **k)** amplitudes and **l)** frequencies across a range of illumination intensities and attached loads at an excitation length of 7 mm. Irradiation conditions: 532 nm. The error bars are displayed as mean values +/- standard deviation (n = 3). The same sample was measured repeatedly.

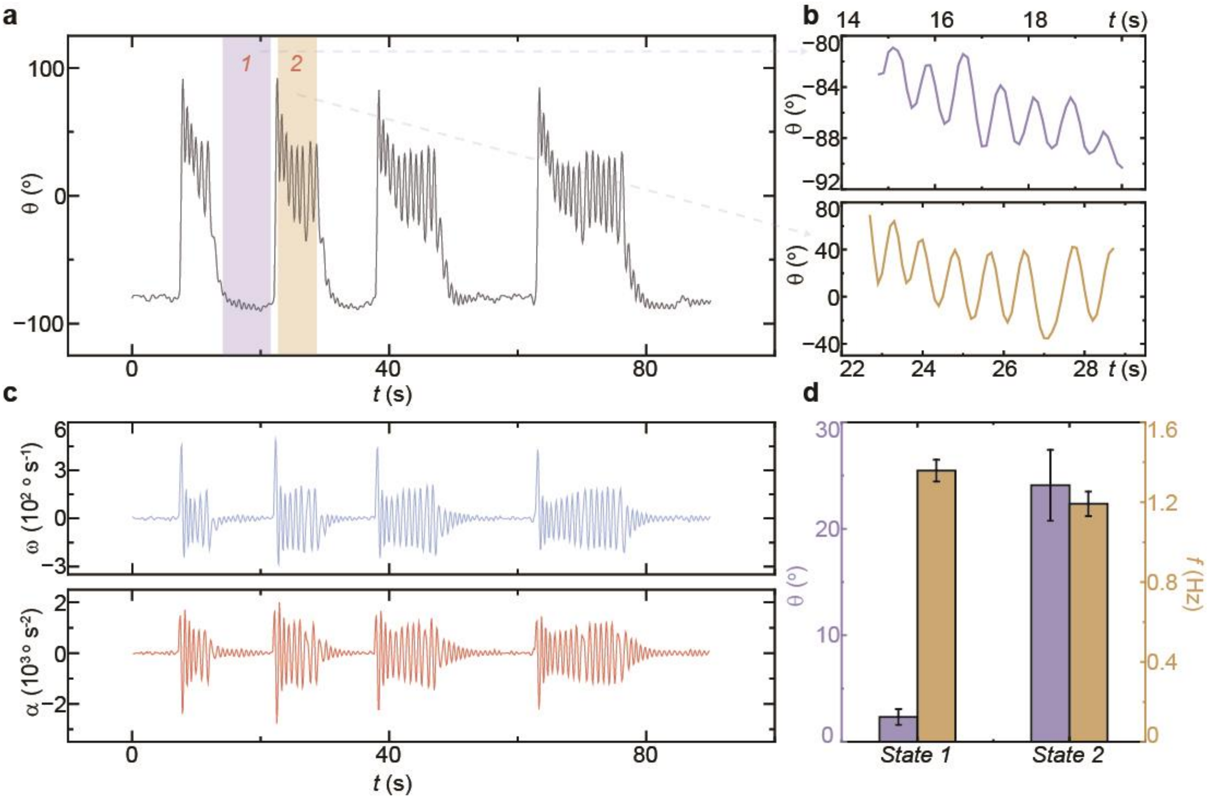

Supplementary Figure 12. Kinetics of spontaneous switching. a) Time-resolved rotation angle of the planar-aligned LCN strip, attached with 0.3 g load, under 167 mW cm^{-2} illumination. Highlighted areas representing for the two distinct oscillation states. **b)** Magnified view of the time-resolved rotation angle of the (top) state 1 and (bottom) state 2. **c)** Corresponding time-resolved of (top) angular velocity and (bottom) acceleration. **d)** Summary of oscillation amplitude and frequency of the two states. Irradiation conditions: 532 nm. The error bars in (d) are displayed as mean values +/- standard deviation (n = 3). The same sample was measured repeatedly.

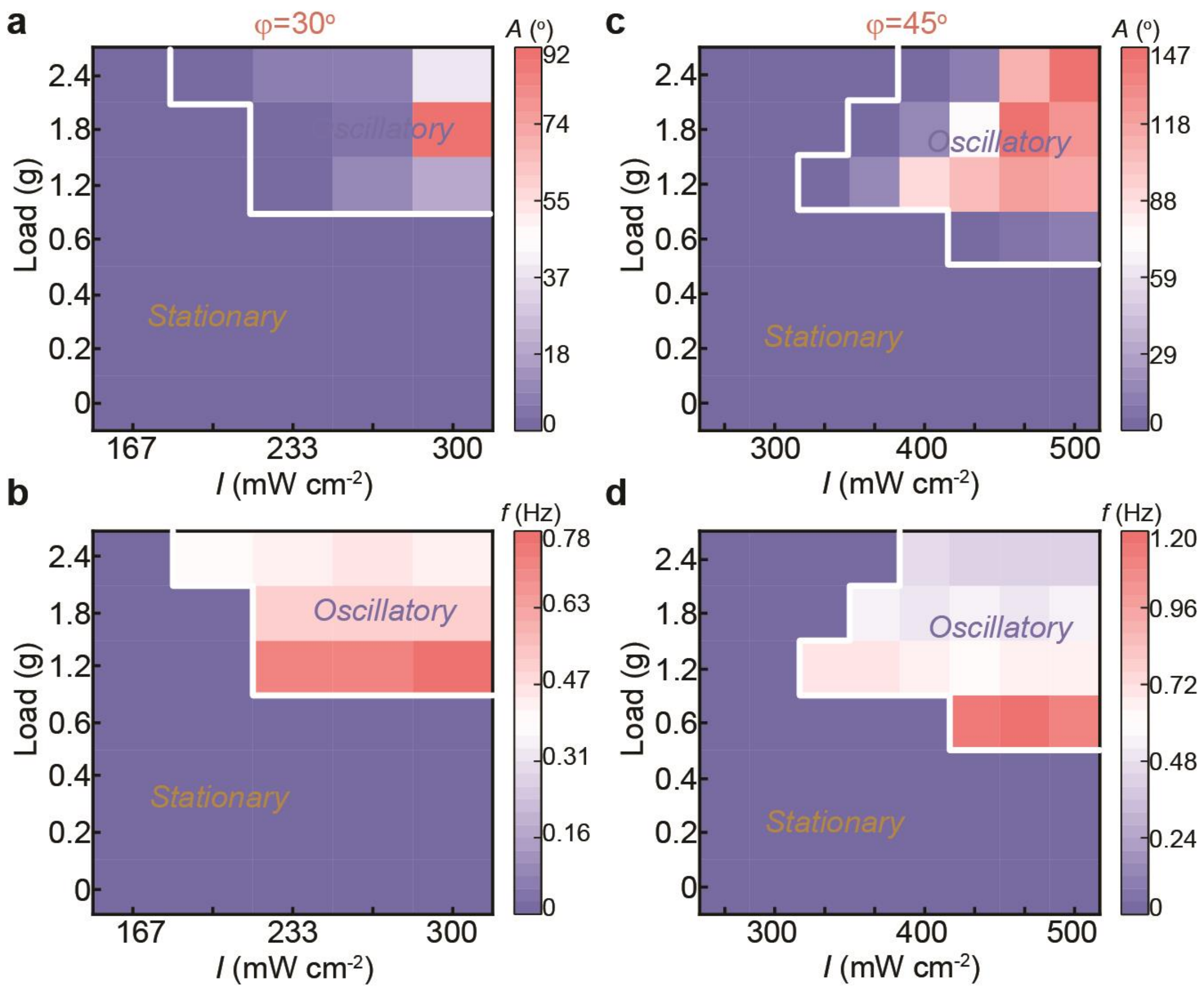

Supplementary Figure 13. Effects of off-axis angle on oscillation in planar mode. Heat map illustrating the oscillation **a)** amplitudes and **b)** frequencies across a range of illumination intensities and attached loads at $\varphi = 30^{\circ}$. Heat map illustrating the oscillation **c)** amplitudes and **d)** frequencies across a range of illumination intensities and attached loads at $\varphi = 45^{\circ}$. Irradiation conditions: 532 nm.

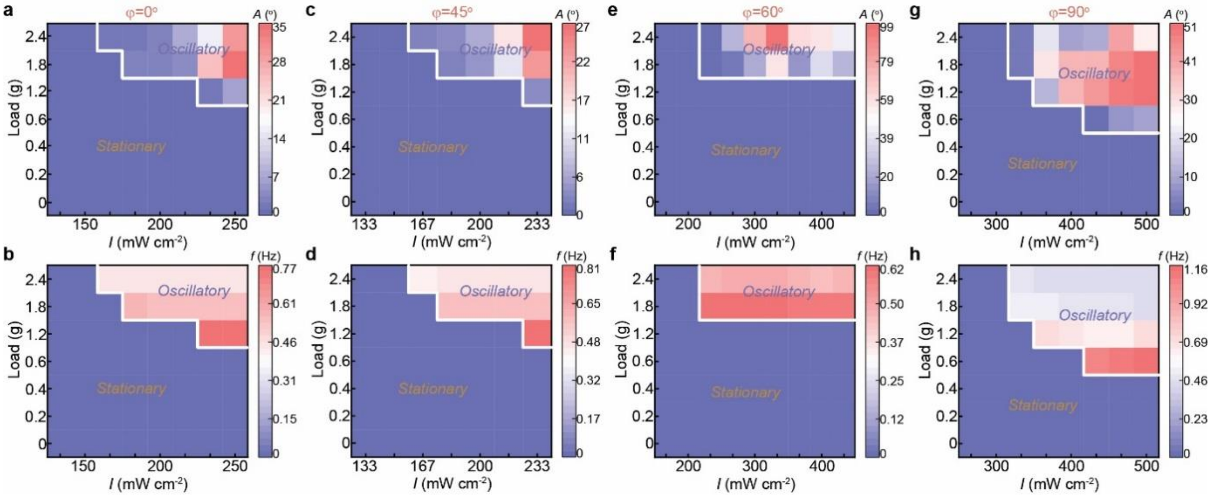

Supplementary Figure 14. Effects of off-axis angle on oscillation in splay mode. Heat map illustrating the oscillation **a)** amplitudes and **b)** frequencies across a range of illumination intensities and attached loads at $\varphi = 0^{\text{o}}$. Heat map illustrating the oscillation **c)** amplitudes and **d)** frequencies across a range of illumination intensities and attached loads at $\varphi = 45^{\text{o}}$. Heat map illustrating the oscillation **e)** amplitudes and **f)** frequencies across a range of illumination intensities and attached loads at $\varphi = 60^{\text{o}}$. Heat map illustrating the oscillation **g)** amplitudes and **h)** frequencies across a range of illumination intensities and attached loads at $\varphi = 90^{\text{o}}$. Irradiation conditions: 532 nm.

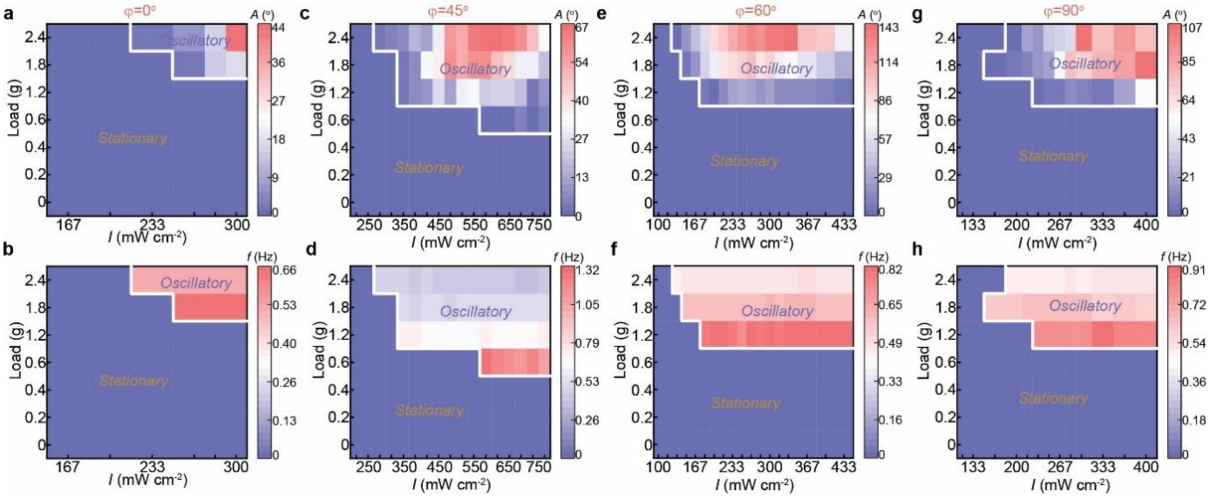

Supplementary Figure 15. Effects of off-axis angle on oscillation in twist mode. Heat map illustrating the oscillation **a)** amplitudes and **b)** frequencies across a range of illumination intensities and attached loads at $\varphi = 0^{\circ}$. Heat map illustrating the oscillation **c)** amplitudes and **d)** frequencies across a range of illumination intensities and attached loads at $\varphi = 45^{\circ}$. Heat map illustrating the oscillation **e)** amplitudes and **f)** frequencies across a range of illumination intensities and attached loads at $\varphi = 60^{\circ}$. Heat map illustrating the oscillation **g)** amplitudes and **h)** frequencies across a range of illumination intensities and attached loads at $\varphi = 90^{\circ}$. Irradiation conditions: 532 nm.

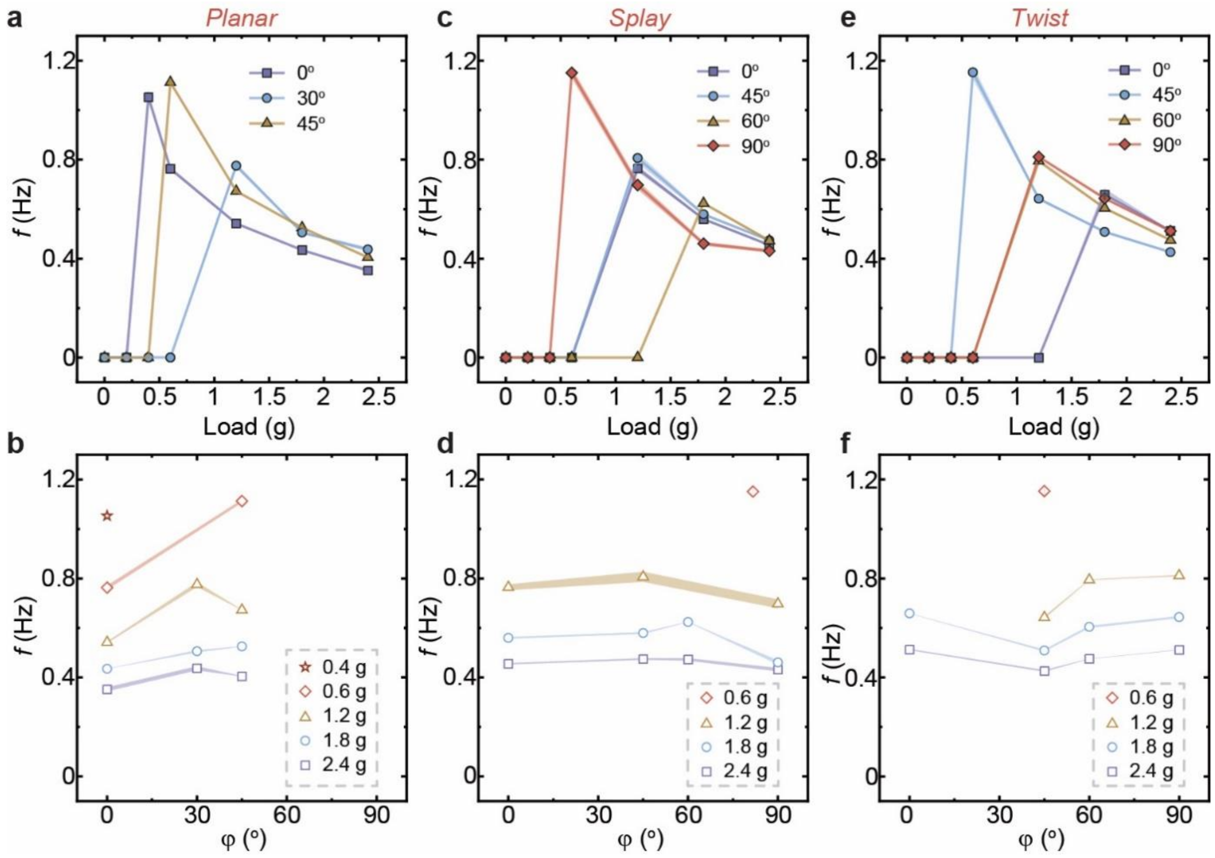

Supplementary Figure 16. Summaries on different modes. Summary of **a)** load effects and **b)** off-axis angle effects on oscillation frequency in planar deformation mode. Summary of **c)** load effects and **d)** off-axis angle effects on oscillation frequency in splay deformation mode. Summary of **e)** load effects and **f)** off-axis angle effects on oscillation frequency in twist deformation mode. Irradiation conditions: 532 nm. The error bars are displayed as mean values +/- standard deviation (n = 3). The same sample was measured repeatedly.

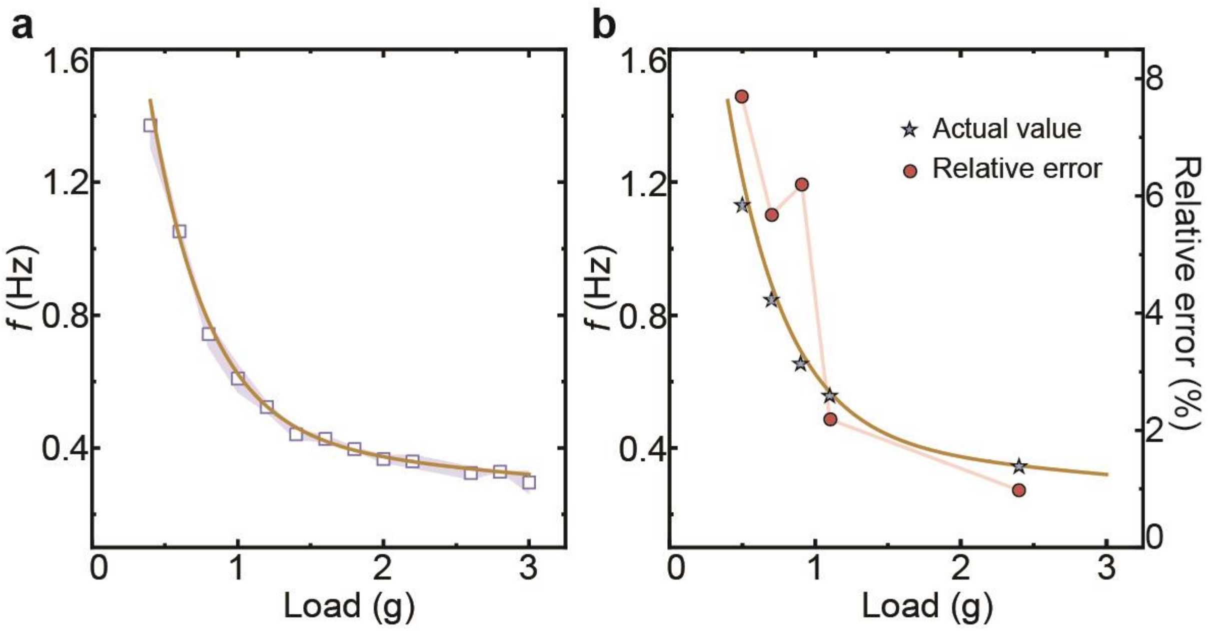

Supplementary Figure 17. Estimation of unknown loads. a) Calibration curve obtained by fitting measured oscillation frequencies for known masses. **b)** Blind test for unknown weights and their corresponding relative errors. $Relative\ error = \frac{|Estimated - Actual\ mass|}{Actual\ mass}$. The error bars are displayed as mean values +/- standard deviation (n = 3). The same sample was measured repeatedly. Irradiation conditions: 532 nm, 200 mW cm^{-2}.

2. Supplementary Movie captions.

Supplementary Movie 1-Load-free shape morphing.

This movie shows LCN strips cut at a 45° off-axis angle undergo shape morphing under load-free condition for three deformation modes.

Supplementary Movie 2-Force field-assisted self-oscillation.

This movie shows LCN strips cut at a 45° off-axis angle undergo tailored self-oscillation dynamics under 1.2 g and 2.4 g load conditions for three deformation modes.
