# Supplementary material for: A light-fueled self-oscillator that senses force
Source: Commun Mater. 2025 Aug 5;6(1):173. doi: 10.1038/s43246-025-00903-2 (PMC12325081; doi:10.1038/s43246-025-00903-2)
Supplement: Supplementary file 3 — Description of Additional Supplementary File [file 43246_2025_903_MOESM3_ESM.pdf]

Description of additional supplementary file

Supplementary Movie 1

Load-free shape morphing. This movie shows LCN strips cut at a 45° off-axis angle undergo shape morphing under loadfree condition for three deformation modes.

Supplementary Movie 2

Force field-assisted self-oscillation. This movie shows LCN strips cut at a 45° off-axis angle undergo tailored self-oscillation dynamics under 1.2 g and 2.4 g load conditions for three deformation modes.
